# Guselkumab is effective in the treatment of Inverse psoriasis: a case report and literature review

**DOI:** 10.3389/fmed.2026.1789385

**Published:** 2026-03-31

**Authors:** Zhuochen Wu, Xiaoyu Xie, Wenyu Tang, Yifeng Wu, Guoqiang Zhang, Qing Zhu

**Affiliations:** 1 Department of Dermatology, The First Hospital of Hebei Medical University, Shijiazhuang, Hebei, China; 2 Subcenter of National Clinical Research Center for Skin and Immune Diseases, Shijiazhuang, Hebei, China; 3 Hebei Technical Innovation Center of Dermatology and Medical Cosmetology Technology, Shijiazhuang, Hebei, China; 4 The Second Hospital of Hebei Medical University, Shijiazhuang, Hebei, China; 5 Affiliated Hospital of Hebei Engineering University, Handan, Hebei, China

**Keywords:** biologics, clinical efficacy, Guselkumab, interleukin-23 inhibitor, Inverse psoriasis

## Abstract

Inverse psoriasis is a special type of psoriasis whose pathogenesis is not yet fully understood, and for which there is no consensus guideline due to marked therapeutic challenges associated with the specific anatomic location of lesions. Immune-mediated inflammation plays a key role in its development, and the lesions are susceptible to friction and humidity, which makes the affected area less tolerant to treatment. In this article, we report a successful case of Inverse psoriasis treated with Guselkumab in a 48-years-old male, who was treated with three subcutaneous injections of Guselkumab and exhibited marked improvement in cutaneous lesions and pruritus at 12 weeks of treatment. A review of the relevant literature was also conducted with the aim of providing clinicians with more references for the treatment of this disease.

## Introduction

Psoriasis is a common chronic inflammatory skin disease, mainly characterized by erythema and scales, with a prolonged course and easy recurrence, which seriously affects patients’ quality of life. In recent years, biological agents have demonstrated significant advantages in the treatment of psoriasis through their precise targeting mechanisms, representing an important trend in current clinical practice (1). As a special subtype of psoriasis, Inverse psoriasis occurs in skin folds, presenting with atypical clinical manifestations and posing challenges for treatment (2). Guselkumab, as a biologic agent targeting interleukin-23, has been shown to have good efficacy in various types of psoriasis, including plaque psoriasis (3). Compared with other IL-23 inhibitors like risankizumab and tildrakizumab, Guselkumab offers unique advantages for Inverse psoriasis. It shows superior efficacy in genital and intertriginous lesions, has a longer half-life to reduce injection frequency and improve compliance–key for long-term maintenance–and causes fewer local injection site reactions, suiting patients with sensitive skin in intertriginous areas (3, 4). Based on this, we attempted to apply it to Inverse psoriasis, with the aim of providing a new idea for the treatment of this particular subtype.

## Case report

The patient, a 48-years-old male, visited the outpatient department of our hospital due to “skin rashes with itching on the head and face, groin, scrotum, and perianal area for 1 year.” One year ago, without obvious inducement, the patient developed erythema and scales on the scalp accompanied by itching. He went to another hospital, where he was diagnosed with seborrheic dermatitis. He was treated with topical mometasone furoate cream, selenium disulfide lotion, and oral methylprednisolone tablets, but the therapeutic effect was unsatisfactory, and the condition was prone to recurrence. Later, the area of erythema gradually expanded, spreading to the groin, scrotum, and perianal area. Subsequently, the patient visited the outpatient department of our hospital, and relevant laboratory tests and pathological biopsy were completed. He had been in good health previously, with no history of chronic diseases including hypertension or diabetes, no drug or food allergy history, and no family history of psoriasis.

**FIGURE 1:**
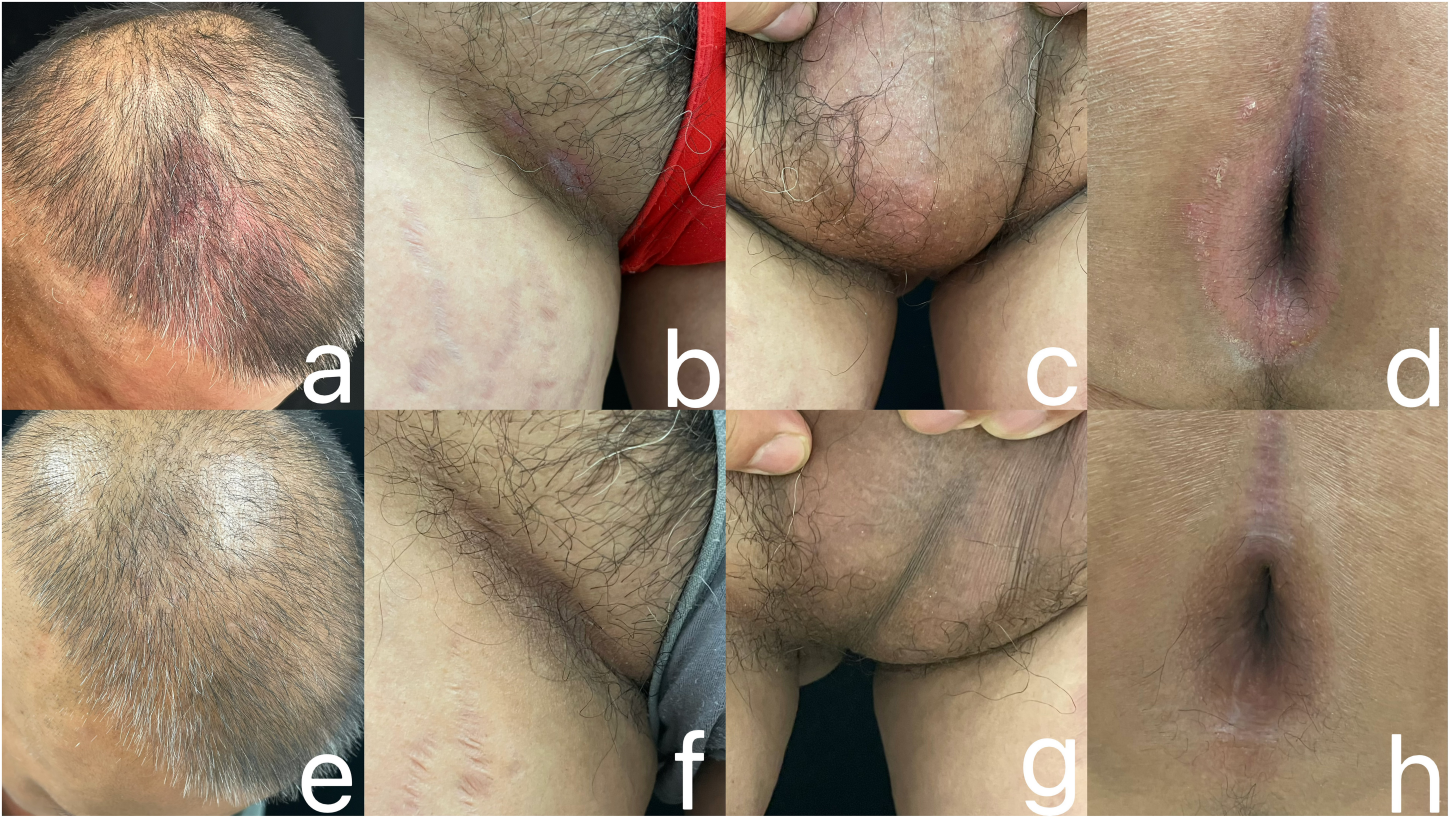
Before treatment, the patient had erythema with a small amount of scaling on the scalp **(a)**, groin **(b)**, scrotum **(c)** and perianal area **(d)**; After receiving 3 doses of Guselkumab, the erythema and scaling on the scalp **(e)**, groin **(f)**, scrotum **(g)** and perianal area **(h)** improved significantly.

**FIGURE 2:**
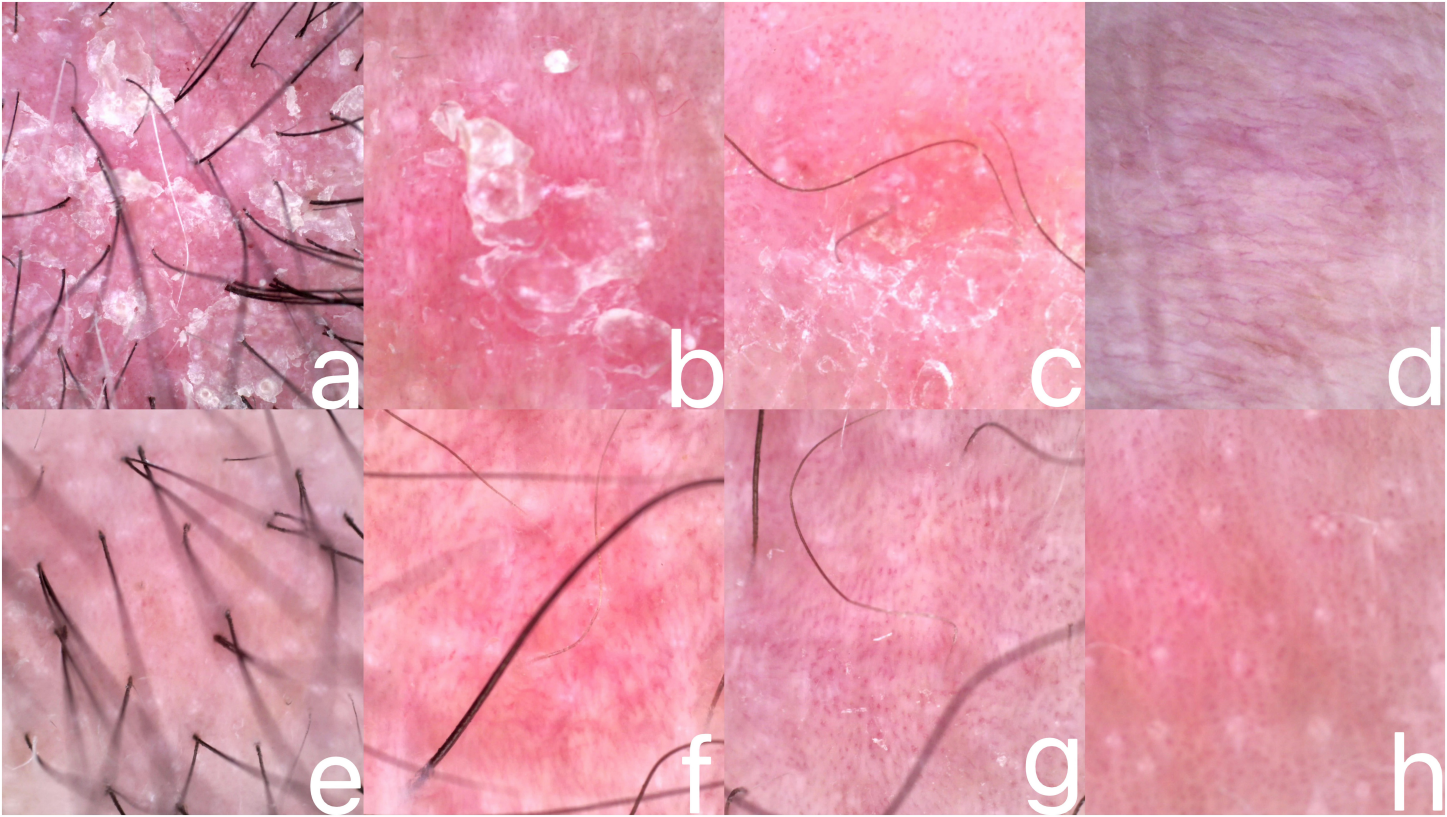
Before treatment, erythema with scaling on the scalp **(a)**, groin **(b)**, scrotum **(c)**, and perianal area **(d)**, with blotches and linear vessels on the margins, vascular disorders at the fusion sites, and vesiculation and oozing in the groin at the epidermal exfoliation sites. After treatment: scalp **(e)**, groin **(f)**, scrotum **(g)**, perianal **(h)** erythema is reduced, scaling is reduced, vascularization is reduced, inguinal vesicles exudate disappears, scrotum flushing and greasy shine is reduced, perianal skin thickening improves.

Dermatology examination showed scattered erythematous plaques with fine desquamation and well-demarcated borders on the head and face, partially fused, visible scratch marks, and a positive Auspitz sign/film sign; a clearly defined red plaque was visible on the right groin, with a smooth and moist surface, epidermal exfoliation accompanied by mild oozing, and a slight sense of infiltration on palpation; diffuse erythema of the scrotum, with a clear border and a moist surface; an annular erythema was noted in the perianal area, with clear borders, and a mild thickening of the skin locally, deepening of the texture, and scattered tiny scales (Figures 1a–d). Dermoscopy showed erythematous, scaly patches visible on the scalp, groin, scrotum, and perianal area, accompanied by various forms of vascularization such as punctate and linear vessels, with vesicular oozing and thickening changes of the skin in some areas (Figures 2a–d).

**FIGURE 3:**
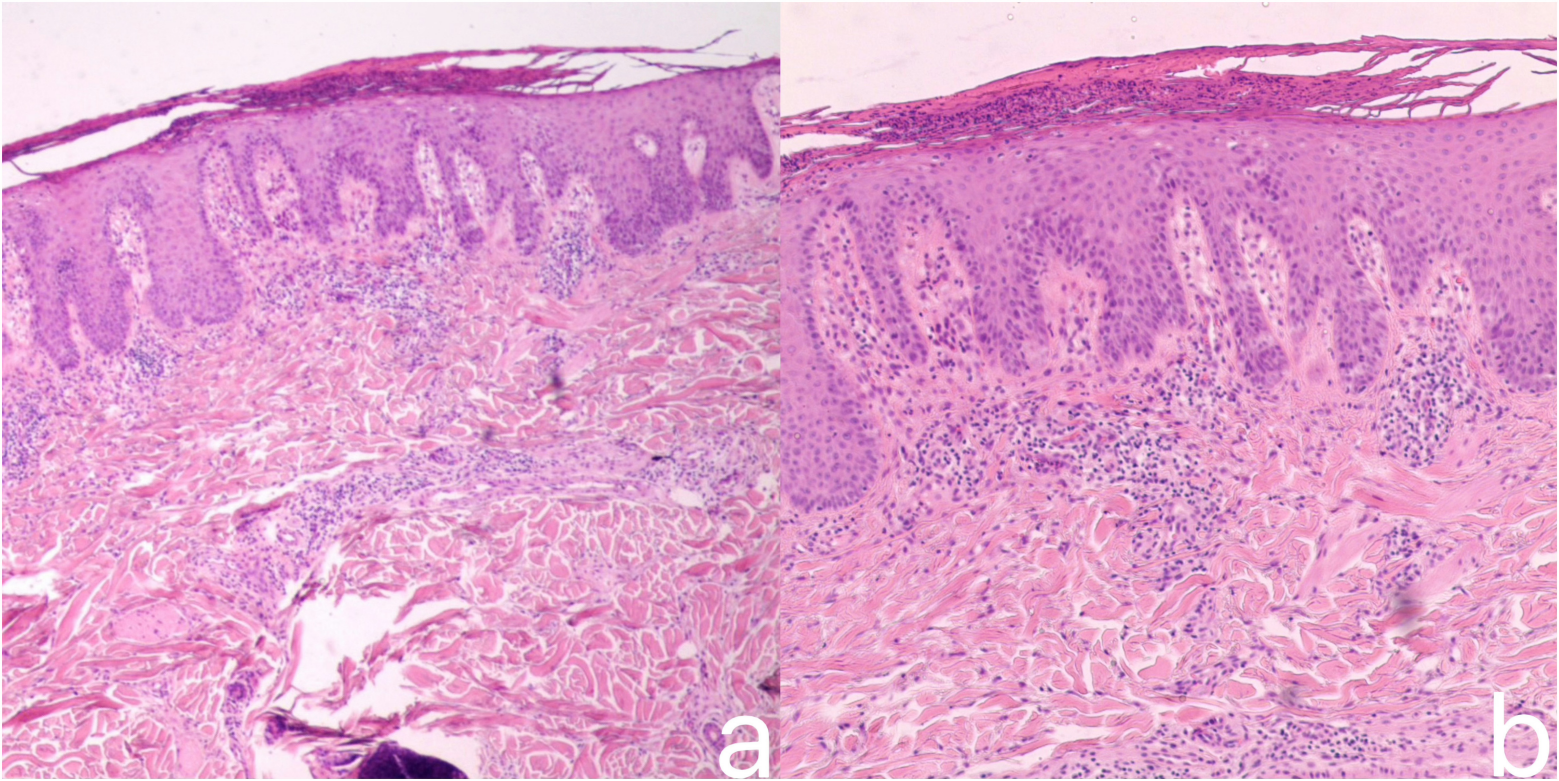
Histopathological examination of the skin showed fused hyperkeratosis, microabscesses within the stratum corneum, irregular hyperplasia of the stratum spinosum, peg-like elongation of the stratum spinosum, intact basal cells, dermal papillae edema, and a small infiltration of lymphocytes and histiocytes around the dermal vasculature and appendages **(a)** H&E, ×40; **(b)** H&E, ×100.

Laboratory tests: complete blood count, urinalysis, liver and renal function tests, coagulation function, blood lipids, blood sugar, and infectious disease tests showed no obvious abnormalities; antinuclear antibody screening was negative; and Fungal β-D-glucan test was negative. Skin histopathological examination showed fused hyperkeratosis, intracorneal microabscesses, irregular hyperplasia and peg-like elongation of the stratum spinosum, intact basal cells, dermal papillae edema, and perivascular and periadnexal eosinophilic infiltrates. Quantitative analysis revealed 42% stratum spinosum thickening, 18 ± 3 eosinophils per high-power field around dermal vessels and appendages, 15% stratum corneum microabscesses relative to total epidermal area, and a dermal papillary edema index of 0.63 as edematous papillae area over total papillae area, all of which were consistent with the typical histopathological features of Inverse psoriasis (5) (Figures 3a, b). The combination of clinical symptoms, dermoscopy and pathologic examination confirmed the diagnosis of Inverse psoriasis.

Considering the poor response to conventional therapy and the patient’s demand for safe and effective treatment, we selected Guselkumab for its unique advantages: as a highly specific IL-23 p19 inhibitor, it exerts superior efficacy in genital and intertriginous lesions with fewer injection site reactions and a longer half-life for better compliance, which is more suitable for sensitive intertriginous skin. Conventional systemic drugs (e.g., methotrexate) were excluded due to their non-specific anti-inflammatory effects and potential systemic adverse reactions requiring strict organ function monitoring. we decided to administer Guselkumab 100 mg subcutaneously, and the same dosage was given again subcutaneously at weeks 4 and 12, and at the 12th week of treatment, the patient’s pruritus was markedly alleviated, and the color of the skin rash became lighter with a reduced size (Figures 1e–h). Dermoscopic examination of the scalp, groin, scrotum and perianal area showed improvement of the lesions, as evidenced by a decrease in erythema and scaling, a reduction in vascular abnormalities, and the disappearance of vesicular ooze (Figures 2e–h). His Dermatology Life Quality Index (DLQI) score decreased from 12 to 4. Standardized assessments for Inverse psoriasis were applied. The Static Physician Global Assessment for Genital Areas (sPGA-G) score fell from 4 to 1 and the Intertriginous Physician Global Assessment iPGA score dropped from 3 to 0 at week 12. DLQI subdomain analysis showed embarrassment reduced from 3 to 0, intimacy impairment from 2 to 0, and daily function limitations from 5 to 1. Safety surveillance included monthly body temperature checks complete blood count and C-reactive protein testing with all results normal white blood cell count 4.5–6.2 × 10^9^/L; C-reactive protein below 5 mg/L and no infections reported. The patient was satisfied with the treatment results and agreed to continue treatment with Guselkumab, and the patient had no adverse events (AEs) including infection or hypersensitivity during treatment. The patient agreed to continue Guselkumab treatment, with the following future management plan: 100 mg subcutaneously every 8 weeks as maintenance dose starting from week 16; follow-up every 3 months with dermatological examination, sPGA-G/iPGA/DLQI assessment, and safety monitoring (blood routine, liver and kidney function) every 6 months; meanwhile, the patient is advised to keep intertriginous skin dry and avoid friction to reduce recurrence risk. Dose adjustment will be considered if lesions achieve complete clearance for 12 consecutive months.

## Discussion

Inverse psoriasis is an uncommon variant of psoriasis that is unique in terms of clinical features, pathogenesis, diagnosis, and treatment. Its prevalence varies widely across studies and populations, ranging from approximately 3%–36% (6).

Inverse psoriasis is most commonly found in skin folds such as the inguinal region (95.8%) (2). This is due to the interaction between the special environment of these areas and the disease mechanism: moist, sweaty, high-temperature, and frequently rubbed fold conditions easily damage the skin barrier and induce inflammation, while the skin here has a weak barrier and dense sensitive immune cells. Environmental stimulation activates local immune responses, amplifying psoriasis-characteristic immune inflammation and abnormal keratinocyte proliferation, ultimately leading to concentrated skin lesions in folds (6, 7). Due to moist and friction-prone conditions, lesions are mostly moist, smooth, and without typical scales, aiding differentiation from other psoriasis types (8–10). Additionally, patients experience a poorer health-related quality of life than those with plaque psoriasis, especially regarding pruritus and shame (11). Diagnosis is based on clinical manifestations, combined with skin biopsy, bacterial/fungal examination to rule out secondary infections or similar rashes, plus evaluation of skin and mucous membranes, nails, joints, and family history; skin biopsy may assist in difficult single-symptomatic cases (8). In this case, the patient’s inguinal, scrotal, and perianal folds showed typical moist, smooth lesions without excessive white scales, providing key clues for initial diagnosis. Dermoscopy, unobscured by scales, facilitated observation of lesion vascular structure details and Inverse psoriasis-specific vascular changes, demonstrating its diagnostic value (12, 13).

The pathogenesis of Inverse psoriasis is similar to that of plaque psoriasis (8), but histopathologically, it often exhibits atypical features including intradermal eosinophilic infiltration, epidermal spongiosis, and focal serous components in the scales (5). In this case, the patient’s skin histopathologic examination showed intradermal eosinophilic infiltration, which is consistent with atypical features of Inverse psoriasis, which confirms the importance of pathologic examination in confirming the diagnosis of Inverse psoriasis.

Treatment for Inverse psoriasis follows a stepwise approach. First-line treatment is mainly based on topical low- to moderate-potency corticosteroids, calcineurin inhibitors (tacrolimus, pimecrolimus), and topical vitamin D analogs (calcitriol) (9, 14–16). Topical retinoids and salicylic acid, excimer laser therapy, and botulinum toxin injections may also be used as adjunctive therapies (17, 18). However, the treatment strategy of Inverse psoriasis differs significantly from that of chronic plaque psoriasis due to the characteristics of thin skin at the lesion site, high sensitivity and mostly in occluded areas, with more focus on the use of low-potency topical steroids or topical immunomodulators in the selection of specific medications. (19). The patient in this case had previously received ineffective conventional treatments such as topical glucocorticoids and was prone to recurrent disease. This exemplifies the limitations of conventional treatments in dealing with Inverse psoriasis.

In recent years, biologics have been increasingly used in the treatment of psoriasis. A small number of cases and studies have confirmed their potential in the treatment of Inverse psoriasis: Ješe et al. showed in his report that a patient with concomitant psoriatic arthritis and severe lesions in the genitals and intergluteal folds had almost complete regression of lesions in the genital area after 90 days of treatment with adalimumab (20); Campos et al. reported that a patient’s psoriasis involving the groin, gluteal fissure, axilla, glans penis, and other parts of the body improved significantly after receiving four injections of ustekinumab (21); Li et al. reported complete regression of skin lesions after treatment with infliximab in a 25-years-old male patient with no recurrence at 3-months follow-up (22); and another study showed that, in a phase 3b randomized controlled trial of ixekizumab for the treatment of Inverse psoriasis, treatment another study showed that in a phase 3b randomized controlled trial of ixekizumab for the treatment of Inverse psoriasis, 73% of patients achieved “clearance” or “substantial clearance” on the Physician’s Global Assessment of the Genital Area (sPGA-G) after 12 weeks of treatment, far exceeding the 8% in the placebo group (9), which opens up new options for patients who have been poorly treated with conventional therapy.

Guselkumab is a fully human monoclonal antibody that selectively binds to the p19 subunit of interleukin-23 (IL-23), blocking the binding of IL-23 to the receptor and inhibiting its biological activity, thus interrupting the downstream inflammatory cascade, attenuating the activation and proliferation of Th17 cells, reducing the secretion of proinflammatory cytokines, such as IL-17 and IL-22, and inhibiting the abnormal proliferation and differentiation of keratinocytes, reduce skin inflammation and erythema scaly symptoms, and then effectively treat psoriasis (4).

Safety is critical for long-term biologic therapy in Inverse psoriasis. Our patient had no adverse reactions over 12 weeks, consistent with recent data. Phase III and real-world analyses demonstrate that Guselkumab carries a low risk of serious infection – comparable to placebo – and a lower incidence of injection site reactions than other IL-23 inhibitors, a profile that is particularly beneficial for patients with sensitive intertriginous skin. To further contextualize its safety in Inverse psoriasis, a comparison with plaque psoriasis using real-world evidence from the large cohorts reported by Hugo et al. and Megna et al. (23, 24) confirms a highly consistent overall adverse event (AE) profile between these two subtypes, characterized by low rates of mild infections and negligible serious AEs. Notably, despite the inherent predisposition of Inverse psoriasis to intertriginous infections, neither study documented a subtype-specific elevation in cutaneous or mucosal infections. Long-term follow-up, alongside these real-world data, validates no major safety concerns for Guselkumab; additionally, the agent exhibits favorable tolerability in biologic-experienced patients and those with comorbidities such as psoriatic arthritis and obesity across both psoriasis subtypes, with no evidence of differential safety signals that would warrant distinct monitoring strategies for patients with Inverse psoriasis.

Here we conducted a systematic review of the literature on Inverse psoriasis treatment regimens and summarized 13 cases to explore the characteristics of the various treatment regimens (Table 1). Literature reports show a wide age range of patients, from children to the elderly, with an age range of 10–65 years, no significant difference in gender, and a duration of disease ranging from 3 months to 8 years. The specific triggering factors of most cases have not been clarified, and only a few of them are related to drugs such as teriflunomide, hydroxychloroquine, or T-cell-mediated autoimmune reactions, which reflects the complexity and diversity of the etiology of this disease, which may involve genetic, immune, environmental, and other factors, and still requires further research and exploration.

**TABLE 1:** Summary of possible causes and treatment of Inverse psoriasis.

References	Age/gender	Disease duration (Inverse psoriasis)	Possible causes	Treatment and outcome
Li et al. (22)	25/man	N/A	N/A	Infliximab Lesions resolved; no recurrence in 3-months follow-up.
Ješe et al. (20)	37/man	Three years	N/A	Adalimumab: near-complete clearance of psoriatic lesions at 90 days. 6-months follow-up: no recurrence or adverse reactions.
Campos et al. (21)	48/man	Two years	N/A	Ustekinumab Significant improvement in pruritus, erythematous lesions, and quality of life.
Mueller et al. (31)	49/woman	Two years	Oral teriflunomide	N/A
Ullah et al. (32)	65/woman	N/A	Oral hydroxychloroquine	Hydroxychloroquine was discontinued Oral methotrexate achieved complete resolution of skin lesions within 1 week.
Wang et al. (25)	10/man	Two years	N/A	Apremilast Significant improvement in itching and lesions
Tajalli et al. (26)	30/man	Eight years	N/A	Tofacitinib Improvement within 1 week and long term remission
Mafong et al. (17)	26/woman	One and a half years	N/A	308 nm excimer laser (spot size 3.5 cm, pulse width 30 ns), twice a week, at least 48 h apart, for 3 weeks (6 treatments). The lesions began to improve after 1 week of treatment, and after 3 weeks (6 treatments) the affected areas had improved by 90% and were close to disappearing completely. There was no recurrence at 6 months of follow-up. During the treatment, the patient only felt a slight warm sensation, and there were no adverse reactions such as blisters, hyperpigmentation, excessive erythema, or pain.
Syed et al. (33)	53/woman	Nine months	T cell-mediated autoimmune response	Treatment with moderate-potency topical glucocorticoids for 2 months The skin lesions basically subsided.
Zheng et al. (28)	26/woman	Three months	N/A	Cold atmospheric plasma, 2–3 times per week The lesions almost completely resolved, with only a brownish-red plaque remaining, and no signs of recurrence were observed during the 6-weeks follow-up.
Zheng et al. (28)	38/woman	Four years	N/A	Cold atmospheric plasma, once every 3 days, 5 min per lesion site, 8 total sessions. The pruritus symptoms were significantly relieved, the lesions subsided markedly, and no recurrence was observed during a 1-month follow-up.
Carrascosa et al. (27)	56/woman	Four years	N/A	308 nm excimer laser twice weekly; after 11 weeks, add tacrolimus ointment twice daily. The patient achieved a 90% improvement in lesions 2 weeks after tacrolimus ointment initiation; excimer therapy stopped. Complete remission in another week on the ointment. Mild inframammary recurrence at 1-month follow-up, controlled with a few days of the ointment.
Wu et al. (29)	9/man	Four years	N/A	Tacrolimus ointment topically plus traditional Chinese medicine bath: 30 g each of Rhubarb, *Salvia miltiorrhiza*, Folium Isatidis, *Albizia julibrissin* (anti-inflammation, blood circulation, stasis removal); 30 g *Hibiscus syriacus* bark (anti-itch); 12 g *Atractylodes lancea* (dampness elimination); 30 g *Polygonatum sibiricum* (yin nourishment, skin hydration). Use: Boil herbs 30 min, soak lesions twice daily, 15 min each. Two weeks later, erythema was significantly alleviated and pruritus resolved completely; all lesions disappeared entirely 1 month later.

In terms of treatment, various methods have shown varying degrees of efficacy. Biological agents and small molecule targeted drugs, such as infliximab, adalimumab, ustekinumab and other biological agents, as well as small molecule targeted drugs, such as apremilast and tofacitinib, can effectively improve the symptoms of lesions and itching, and in some cases, long-term remission or no recurrence can be achieved, which provides an important option for patients with poor results of traditional treatment, such as Wang et al. reported that a 10-years-old male patient achieved significant pruritus relief and lesion improvement after apremilast treatment. Wang et al. reported that a 10-years-old male patient with apremilast showed significant improvement in itching and skin lesions (25), and Tajalli et al. reported that tofacitinib showed improvement within 1 week of treatment in a 30-years-old male case and long-term remission was achieved, suggesting that JAK inhibitors may offer a new option for Inverse psoriasis (26). In physical therapy, 308 nm excimer laser alone or in combination with topical immunomodulators such as tacrolimus ointment showed significant improvement in lesions with fewer adverse effects and a low recurrence rate at 6-months follow-up, suggesting that the combination of physical therapy and topical medications may enhance the efficacy of the treatment, such as in the case of a 26-years-old female patient who was treated with 308 nm excimer laser therapy twice weekly for 3 weeks (6 treatments), as reported by Mafong et al. For example, Mafong et al. reported that a 26-years-old female patient was treated with 308 nm excimer laser twice a week for 3 weeks (6 treatments), and the lesions started to improve after 1 week, and achieved a 90% improvement after 3 weeks with near-complete resolution of lesions; no recurrence was observed during the 6-months follow-up, and the patient only experienced a mild warm sensation, and there were no adverse reactions such as blisters and hyperpigmentation. 90% improvement, complete remission 1 week after discontinuation of the drug, and only mild recurrence under the breast at 1-month follow-up, which was stabilized after short-term control with ointment (27).

In addition, new therapeutic methods also have certain application value. Cold atmospheric plasma can relieve itching and promote the regression of skin lesions; integrated traditional Chinese and Western medicine therapies, such as tacrolimus ointment combined with traditional Chinese medicine baths, are safe and effective for children, and can quickly improve symptoms and eliminate skin lesions. Zheng et al. reported two cases where skin lesions resolved and no recurrence occurred after treatment with cold atmospheric plasma (28); Wu et al. reported one case where the skin lesions were cured after treatment with a traditional Chinese medicine bath combined with tacrolimus ointment (29).

For the specific subtype of Inverse psoriasis, its specific therapeutic efficacy and safety still lack sufficient evidence-based medical evidence support, and further in-depth studies are urgently needed to validate (30). The successful treatment of this case provides a new clinical rationale for the use of Guselkumab in the treatment of Inverse psoriasis, but more clinical studies are still needed to further evaluate its efficacy and safety.

## Conclusion

Inverse psoriasis presents unique clinical features, poses great therapeutic challenges, and lacks standardized evidence-based treatment protocols. In this case, after 12 weeks of treatment with Guselkumab, the patient’s lesions and itching in the head, face, groin, and other parts of the body improved significantly, and the DLQI decreased without adverse effects, demonstrating a positive therapeutic potential. This provides a new idea and reference for clinicians to deal with similar patients who are not well treated with conventional therapy. In view of the small number of relevant studies, multicenter, large-sample, long-term follow-up clinical trials are needed to further validate the long-term efficacy and safety of this treatment and to optimize the treatment strategy.

## Data Availability

The original contributions presented in this study are included in this article/supplementary material, further inquiries can be directed to the corresponding authors.
